# Impact of Continuous Erythropoietin Receptor Activator on Selected Biomarkers of Cardiovascular Disease and Left Ventricle Structure and Function in Chronic Kidney Disease

**DOI:** 10.1155/2016/9879615

**Published:** 2016-02-29

**Authors:** Piotr Bartnicki, Jacek Rysz, Beata Franczyk, Zbigniew Baj, Ewa Majewska

**Affiliations:** ^1^Department of Nephrology, Hypertension and Family Medicine, Medical University of Lodz, 90-549 Lodz, Poland; ^2^Department of Pathophysiology and Clinical Immunology, Medical University of Lodz, 91-647 Lodz, Poland

## Abstract

*Background*. Cardiovascular morbidity and mortality are very high in patients with chronic kidney disease (CKD). The purpose of this study is to evaluate the impact of continuous erythropoietin receptor activator (CERA) on selected biomarkers of cardiovascular disease, left ventricle structure, and function in CKD.* Material and Methods*. Peripheral blood was collected from 25 CKD patients before and after CERA treatment and 20 healthy subjects. In serum samples, we assessed inflammatory markers (IL-1*β*, TNF-RI, TNF-RII, sFas, sFasL, MMP-9, TIMP-1, and TGF-*β*1), endothelial dysfunction markers (sE-selectin, sICAM-1, and sVCAM-1), and volume-related marker (NT-proBNP). All subjects underwent echocardiography and were evaluated for selected biochemical parameters (Hb, creatinine, and CRP).* Results*. Evaluated biomarkers and echocardiographic parameters of left ventricle structure were significantly increased but left ventricle EF was significantly decreased in CKD patients compared to controls. After CERA treatment, we observed a significant increase of Hb and left ventricle EF and a significant decrease of NT-proBNP and MMP-9. There was a significant negative correlation between Hb and TNF-RI, sICAM-1, and IL-1*β*.* Conclusions*. Our results indicate that selected biomarkers related to cardiovascular risk are significantly increased in CKD patients compared to controls. CERA treatment has anti-inflammatory action, diminishes endothelial dysfunction, and improves left ventricle function in these patients.

## 1. Introduction

Cardiovascular diseases (CVD) are significant causes of morbidity and mortality all over the world [1]. Well-known traditional risk factors of CVD are diabetes, hypertension, obesity, and hyperlipidemia. These factors do not fully explain the increase of CVD in patients with chronic kidney disease (CKD) [2] and a very high risk of cardiovascular death, especially in later stages of CKD [3, 4]. Literature data indicate that pathology, manifestations, complications, and management of CVD differ in CKD patients. Standard therapeutic interventions targeted at traditional risk factors of CVD, that are successful in the general population, are ineffective to lower CVD events and mortality in CKD [5]. It is thought that nontraditional risk factors of CVD play a very important role in pathogenesis of premature atherosclerosis and cardiovascular complications in these patients [2]. The most common nontraditional risk factors of CVD in CKD patients are anemia, calcium/phosphorus disorders, hyperparathyroidism, and malnutrition. It is possible that early therapeutic approaches targeting traditional and nontraditional CVD risk factors may prevent CVD events in CKD, but the information concerning CVD, biomarkers, and treatment in early stages of CKD is limited. Recent studies indicate the role of chronic inflammation, oxidative stress, endothelial dysfunction, and immune cell apoptosis in the pathogenesis of accelerated atherosclerosis and CVD in CKD patients [6, 7]. Some biomarkers of chronic inflammation such as interleukin 1 beta (IL-1*β*), tumor necrosis factor-alpha (TNF-*α*), oxidative stress (reactive oxygen species, antioxidant enzymes), endothelial dysfunction (asymmetric dimethylarginine, soluble forms of adhesion molecules), and apoptosis (Fas, Fas ligand) are well known [8–12]. For better understanding of the pathogenesis of CVD in CKD it is necessary to look for new biomarkers. Recent studies have proposed soluble TNF receptor type II (TNF-RII) and tissue inhibitor of metalloproteinase-1 (TIMP-1) as new biomarkers of chronic inflammation [13] and metalloproteinase-9 (MMP-9), soluble Fas (sFas), soluble Fas ligand (sFasL), soluble intercellular adhesion molecule-1 (sICAM-1), and transforming growth factor-beta 1 (TGF-*β*1) as new markers of CVD risk in CKD patients [14–16]. A very important, nontraditional CVD risk factor in CKD patients is anemia [17]. Especially in the later stages of CKD, anemia reduces the quality of life and may increase oxidative stress, endothelial dysfunction, and immune cell apoptosis and consequently may be involved in pathogenesis of premature atherosclerosis and CVD [18]. Anemia treatment with erythropoietin (EPO) in CKD patients seems to have a pleiotropic effect and may reduce chronic inflammation, oxidative stress, and immune cell apoptosis [19]. Our latest studies showed that anemia treatment with methoxy polyethylene glycol-epoetin beta (CERA), a long half-life EPO, in CKD patients may inhibit oxidative stress and immune cell apoptosis [20, 21]. Finally in consequence of these pleiotropic EPO effects it is thought that timely correction of anemia may lead to a significant improvement in the cardiovascular outcome in CKD patients [22]. The purpose of this study was to evaluate the impact of CERA treatment by using methoxy polyethylene glycol-epoetin (MPG-EPO) beta on selected CVD risk biomarkers, especially of inflammation and endothelial dysfunction, and left ventricle structure and function, in nondialyzed CKD patients.

## 2. Materials and Methods

### 2.1. Patient Population

Thirty-five patients (20 men and 15 women, median age 59 years, range 45–69) with CKD were enrolled in the study. All patients were in stage IV of CKD with an estimated glomerular filtration rate (eGFR) of 15–29 mL/min, calculated according to the Modification of Diet in Renal Disease (MDRD) equation. They were on conservative treatment and had never undergone dialysis. Patients with age over 70 years, diabetes, blood transfusion in the past 3 months, acute infection, chronic infection (hepatitides B and C), autoimmune disease, immunosuppressive therapy, increased C-reactive protein (CRP) over 25 mg/L, or a history of malignancy were excluded from the study. The relatively small number of patients, due to many exclusion criteria, was sufficient to give statistical power (70–100%). Causes of CKD were primary hypertension with chronic kidney disease (40%), chronic tubule-interstitial nephritis (23%), chronic glomerulonephritis (20%), and polycystic kidney disease (17%).

The control group included 20 healthy volunteers (12 men and 8 women, median age 56, range 48–63) without CKD (normal eGFR, hemoglobin concentration, urine analysis, and kidney ultrasound). All investigated subjects had not been smoking for at least 5 years.

All CKD patients had anemia with hemoglobin (Hb) concentration lower than 10 g/dL. After exclusion of bleeding, iron deficiency, hemolysis, infection, and severe secondary hyperparathyroidism, they received a subcutaneous injection of MPG-EPO (Mircera, Roche, Basel, Switzerland) in a dose of 0.6 *μ*g/kg once monthly. Treatment was continued until reaching the target Hb concentration of 11–12 g/dL, achieved by 25 patients, who were enrolled in the next part of the study. Ten patients started dialysis treatment before they achieved target Hb and were excluded from the study. Average treatment time was 227 days (from 108 to 428 days), and the average MPG-EPO dose was 50 *μ*g/month (from 30 to 75 *μ*g).

The study was approved by the Ethics Committee of Research of the Medical University of Lodz, number RNN/97/09/KB. Only patients who signed informed consent were included in the study.

### 2.2. Measurement of Biochemical Parameters and Biomarkers

Peripheral blood was drawn from CKD patients twice (before treatment with MPG-EPO and after achieving target Hb concentration) and from healthy volunteers once. Overnight fasting venous blood was collected into sodium citrate tubes and centrifuged at 4°C with a speed of 1000 g for 20 minutes. The obtained serum samples for biomarker assessment were frozen and stored at −80°C until processed. We measured serum concentration of IL-1*β*, soluble TNF receptors I and II (TNF-RI, TNF-RII), sFas, sFasL, soluble endothelial leukocyte adhesion molecule-1 (sE-selectin), sICAM-1, soluble human vascular cell adhesion molecule-1 (sVCAM-1), MMP-9, TIMP-1, and TGF-*β*1 using commercially available human Quantikine ELISA Kits (R&D Systems, Minneapolis, USA). Sample collection and storage, reagent preparation, assay procedure, and calculation of results were performed according to the manufacturer's instructions. The mean minimum detectable doses of assessed biomarkers, according to the manufacturer, were as follows: IL-*β*1, less than 1 pg/mL; sTNF-RI, 0.77 pg/mL; sTNF-RII, 0.6 pg/mL; sFas, 20 pg/mL; sFasL, 2.6 pg/mL; sE-selectin, 0.009 ng/mL; sVCAM-1, 0.6 ng/mL; sICAM-1, 0.096 ng/mL; MMP-9, 0.156 ng/mL; TIMP-1, less than 0.08 ng/mL; TGF-*β*1, 4.61 pg/mL. The biochemical parameters, serum Hb, creatinine, C-reactive protein (CRP), N-terminal pro-brain natriuretic peptide (NT-proBNP) concentration, were assessed using standard techniques in a local laboratory.

### 2.3. Evaluation of CVD

All CKD patients were diagnosed with hypertension and received the following hypotensive drugs: ACE inhibitors, AT-II receptor blockers, loop diuretics, beta blockers, and calcium channel blockers. The majority of them were diagnosed with chronic coronary heart disease and underwent percutaneous coronoplasty with stent implantation. Some of them presented clinical symptoms of chronic heart failure, such as shortness of breath, tiredness, and peripheral edema. To evaluate left ventricle structure and function, echocardiography was performed twice in the CKD patients (before MPG-EPO treatment and after achieving target Hb concentration) and once in the control group. Echocardiographic examination was performed in accordance with the recommendations of the ESC Section of Echocardiography of 2009 using an Aloka ProSound Alpha camera 10 (Hitachi Aloka Medical, Ltd., Tokyo, Japan) by an experienced cardiologist. Measurements were made in the M-dimensional and two-dimensional 2D presentation. Flow parameters were measured by Doppler: continuous wave (CW), pulse method (PM), and tagged color methods and tissue Doppler imaging. The following echocardiographic parameters were assessed: intraventricular septal diameter (IVSd), left ventricular end systolic diameter (LVESd), left ventricular end diastolic diameter (LVEDd), and left atrial diameter (LA). These measurements were used to evaluate left ventricular ejection fraction (EF), left ventricular mass (LVM), left ventricular hypertrophy (LVH), and left ventricular diastolic dysfunction (LVDD). Diastolic function was assessed by determining the velocities of early (E) and late (A) diastolic transmitral flow, the ratio E-to-A (E/A), deceleration time (DT), isovolumic relaxation time (IVRT), and pulmonary vein flow velocities.

### 2.4. Statistical Analysis

Data are presented as the median (Me) and interquartile range (Me; 25–75%). Evaluation of statistical significance was performed with the Wilcoxon signed ranks test for paired data and the Mann-Whitney *U* test for unpaired data. Correlation between Hb concentration, evaluated biomarkers, and echocardiographic parameters was performed by calculation of Spearman *r* correlation coefficient values. Statistical significance was assumed at a* p* value < 0.05. The statistical analysis was carried out using the statistical software Statistica (StatSoft, Inc., Tulsa, OK, USA).

## 3. Results

The results concerning biochemical parameters in CKD patients and the control group are shown in Table 1. Hb concentration and eGFR were significantly lower in CKD patients in comparison to the control group, but serum creatinine, CRP, and NT-proBNP concentrations were significantly higher in CKD patients in comparison to the control group. After MPG-EPO treatment, CKD patients had a significantly higher Hb concentration and significantly lower serum NT-proBNP concentration in comparison to before treatment. MPG-EPO treatment did not change kidney function or serum CRP concentration.

**Figure 1:**
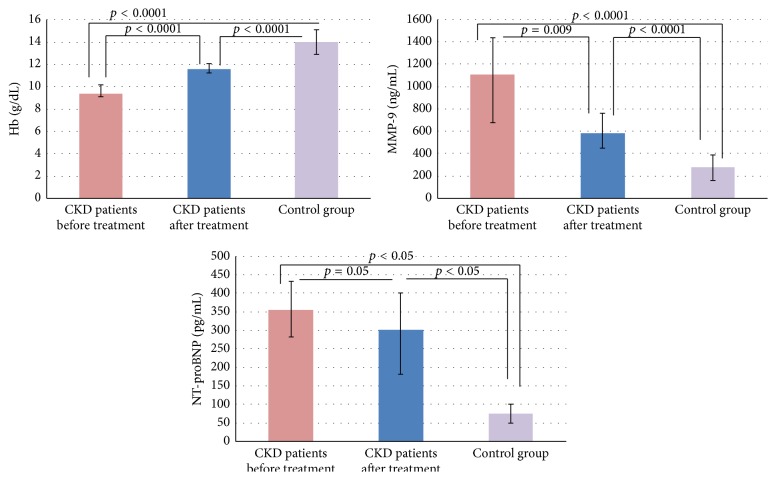
Results of serum Hb, MMP-9, and NT-proBNP concentrations in CKD patients and control group. Hb = hemoglobin, MMP-9 = metalloproteinase-9, NT-proBNP = N-terminal pro-brain natriuretic peptide, and CKD = chronic kidney disease.

The results of evaluated biomarkers in CKD patients before and after MPG-EPO treatment and the control group are shown in Table 2 and Figure 1. Serum concentration of all assessed biomarkers was significantly higher in CKD patients in comparison to the control group. After MPG-EPO treatment, CKD patients had significantly higher serum TNF-RII and sVCAM-1 concentrations, but MMP-9 concentration was significantly lower than before treatment.

**Figure 2:**
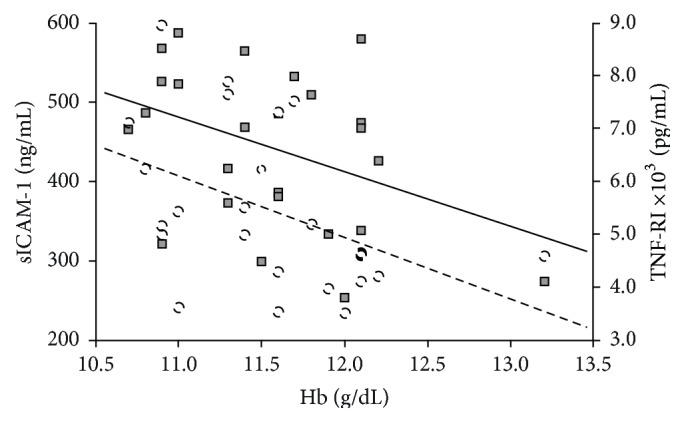
Correlation of serum Hb with TNF-RI and sICAM-1 concentrations in CKD patients. sICAM-1: squares and continuous line; TNF-RI: circles and broken line. Hb = hemoglobin, TNF-RI = soluble tumor necrosis factor receptor I, sICAM-1 = soluble intercellular adhesion molecule-1, and CKD = chronic kidney disease.

Table 3 and Figure 2 show the correlation between Hb concentration and evaluated biomarkers in CKD patients before and after MPG-EPO treatment. There was a significant negative correlation between Hb concentration with serum TNF-RI and sICAM-1 concentrations.

**Figure 3:**
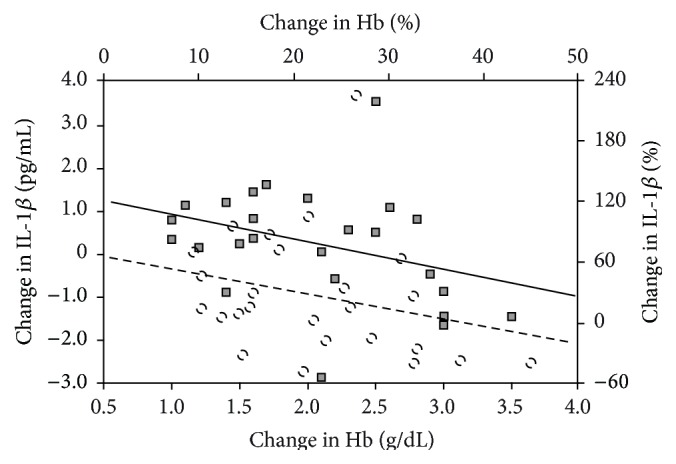
Correlation between increase of Hb concentration expressed as absolute value (g/dL) or as percentage (%) and change of plasma IL-1*β* concentration expressed as absolute value (pg/mL) or as percentage (%) in CKD patients. IL-1 beta (pg/mL) and Hb (g/dL): squares and continuous line; IL-1 beta (%) and Hb (%): circles and broken line. Hb = hemoglobin, IL-1*β* = interleukin 1 beta, and CKD = chronic kidney disease.

The correlations between increase of Hb concentration expressed as absolute values or as percentages and changes of evaluated biomarkers expressed as absolute values or as percentages in CKD patients are shown in Table 4 and Figure 3. There was a significant negative correlation between increase of Hb concentration and serum IL-1*β* concentration.

Evaluated echocardiographic parameters in the study groups are shown in Table 5. All CKD patients had left ventricular hypertrophy (LVH), and the majority of them had left ventricular diastolic dysfunction (LVDD). Left ventricular ejection fraction (EF) was significantly lower in CKD patients than the control group. After MPG-EPO treatment, EF in CKD patients was significantly increased in comparison to before treatment but was still significantly lower than in the control group. Other evaluated echocardiographic parameters of left ventricle structure were significantly higher in CKD patients than in the control group, and MPG-EPO treatment did not change them significantly.

**Figure 4:**
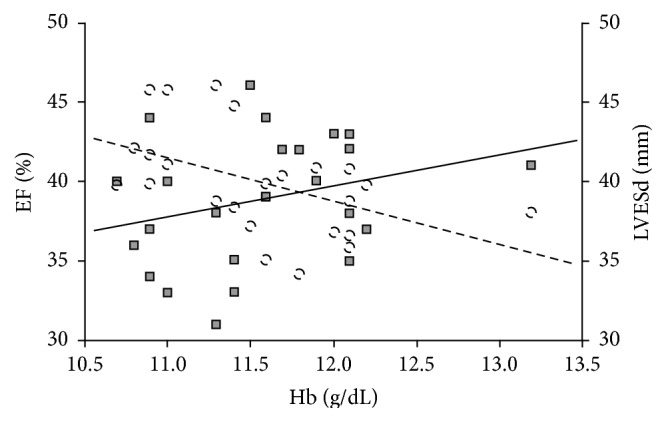
Correlation of serum Hb with EF and LVESd in CKD patients. EF (%): squares and continuous line; LVESd: circles and broken line. Hb = hemoglobin, EF = left ventricular ejection fraction, LVESd = left ventricular end systolic diameter, and CKD = chronic kidney disease.

Table 6 and Figure 4 show the correlation between Hb concentration and evaluated echocardiographic parameters of left ventricle structure and function. There was a significant positive correlation between Hb and EF and a significant negative correlation between Hb and LVESd.

## 4. Discussion

IL-1 (IL-1*α* and IL-1*β*) plays a central role in acute and chronic inflammation [23]. In our study, plasma concentration of the proinflammatory cytokine IL-1*β* was significantly higher in CKD patients than in the control group. Our result is comparable to data available in the literature where the plasma IL-1*β* concentration was found elevated in nondialyzed CKD patients as well as in CKD patients on dialysis treatment [24, 25]. It is well documented that CKD is associated with chronic inflammation and uremic toxins can induce production of IL-1 mainly by monocytes and macrophages [26]. In CKD patients, other proinflammatory cytokines were found to be elevated, such as IL-6 and TNF-*α*, which modify inflammatory and immune reactions [27]. The next step in the inflammatory reaction is increasing expression of cell adhesion molecules (E-selectin, ICAM-1, VCAM-1), responsible for extravasation of leukocytes and activation of metalloproteinases (MMPs). We found in our study significantly elevated plasma sE-selectin, sICAM-1, sVCAM-1, and MMP-9 concentrations in CKD patients in comparison to the control group. Data available in the literature are comparable; Bonomini et al. found elevated serum levels of soluble ICAM-1, VCAM-1, and E-selectin in both nondialyzed CKD patients and those under dialysis treatment [28]. Other authors reported elevated levels of these cell adhesion molecules in patients undergoing chronic hemodialysis [11]. High serum concentration of soluble adhesion molecules in CKD patients may indicate vascular endothelial cell activation, which is involved in the pathogenesis of atherosclerosis and CVD in these patients [29]. Elevated plasma levels of sICAM-1 were strongly associated with CVD and could be considered as a novel biomarker of CVD [30]. Data in the literature regarding serum MMP-9 concentration, where the authors found significantly lower MMP-9 concentration in CKD patients in comparison to the control group [31], are opposite to our results. IL-1 and other proinflammatory cytokines induce synthesis of TIMP-1, an inhibitor of MMP-9, which we found significantly elevated in CKD patients in comparison to the control group. Similar data have been reported by other authors [14, 31]. The latest data in the literature indicate elevated serum MMP-9 concentration as a new biomarker of CVD in CKD [2] and TIMP-1 concentration as a new marker of inflammation in CKD patients [13]. In our study, plasma TGF-*β*1 concentration was significantly higher than in the control group. TGF-*β*1 is well known as a promoter of extracellular matrix synthesis. In experimental chronic renal failure, a correlation was found between elevated levels of TGF-*β*1 and TNF-*α* with cardiac fibrosis [15], which may indicate the involvement of these biomarkers in pathogenesis of CVD in CKD. The next proinflammatory cytokine is TNF, serum concentration of which is elevated in CKD. Literature data indicate that TNF elevation correlates with high concentration of soluble TNF receptors (TNF-RI and TNF-RII), which are thought to be novel biomarkers of inflammation in CKD, especially TNF-RII [2]. We found significantly higher serum TNF-RI and TNF-RII concentrations in CKD patients in comparison to the control group. Similar data were obtained by other authors in nondialyzed CKD patients and in patients under chronic hemodialysis treatment [32]. The Fas/FasL system is related to the endothelial cell apoptosis and inflammatory responses in atherosclerotic plaques [33]. Soluble forms of this system (sFas and sFasL) are proposed as novel biomarkers of vascular damage and high cardiovascular risk. Blanco-Colio et al. showed that increased sFas and decreased sFasL were connected with high cardiovascular risk [16]. In our study, plasma sFas and sFasL concentrations were significantly higher in CKD patients in comparison to the control group. The results of our study indicate that CKD is strongly associated with inflammation, immune system dysregulation, endothelial dysfunction, and vascular damage. All these are involved in pathogenesis of accelerated atherosclerosis and could be responsible for the high rate of CVD in patients with CKD. Patients with CKD enrolled in our study had CVD such as hypertension, chronic coronary heart disease, and chronic heart failure. They had elevated serum NT-proBNP concentration and disorders of left ventricle structure and function in comparison to the control group. Erythropoiesis-stimulating agents (ESAs) are widely used in CKD patients to correct anemia. It is documented in the literature that ESAs beside their main action could have a pleiotropic effect on various cells [34]. It was shown that ESAs could have anti-inflammatory, antioxidant, and antiapoptotic action [19–21]. Anemia correction during ESA treatment can improve heart function [35]. In our study, CKD patients after MPG-EPO treatment achieved a target Hb concentration which was significantly higher in comparison to before treatment. We observed improvement of left ventricle function in CKD patients after MPG-EPO treatment as reflected in a significant increase of EF and a significant decrease of serum NT-proBNP concentration. Echocardiographic parameters of left ventricle structure did not change significantly after treatment, which may be connected with the relatively short average treatment time. Further analysis of the correlation between Hb and echocardiographic parameters of left ventricle structure and function showed a significant positive correlation between Hb and EF and a significant negative correlation between Hb and LVESd. These results indicate that CERA treatment may improve left ventricle function in CKD patients. After MPG-EPO treatment, we observed significantly increased serum TNF-RII and sVCAM-1 concentrations and a significantly decreased serum MMP-9 concentration. The results concerning TNF-RII and sVCAM-1 are unclear. On the one hand, they may indicate that anemia treatment with MPG-EPO enhances inflammation and endothelial dysfunction. We found only one paper in the available literature concerning conversion from short half-life ESAs to MPG-EPO, where the authors noted a significantly increased plasma VCAM concentration after conversion to MPG-EPO (Mircera) [36]. On the other hand, further analysis of our results showed a significant negative correlation between Hb concentration and serum TNF-RI as well as sICAM-1 concentration after MPG-EPO treatment. A significant negative correlation between increase of Hb concentration and change of serum IL-1*β* concentration was also found. Our results concerning serum concentrations of MMP-9, TNF-RI, sICAM-1, and IL-1*β* indicate that CERA treatment using MPG-EPO beside the main action to correct anemia can improve left ventricle function, demonstrate anti-inflammatory action, and may improve endothelial function in nondialyzed CKD patients. In the available literature, most data about ESAs concern the short half-life ESAs used mainly 1–3 times weekly in CKD patients. It is well documented that short half-life ESAs can prevent accelerated atherosclerosis and cardiovascular complications in CKD [19, 35]. However, high doses of the short half-life ESAs were associated with elevated inflammatory biomarkers [37] and higher mortality risk [38]. In our study, we used the long half-life ESA MPG-EPO (CERA), which can be injected once or twice monthly only [39]. We achieved long and slow action of the used ESA with slow correction of anemia, without impairment of renal function and at the same time a positive impact on the evaluated biomarkers of cardiovascular risk and left ventricle function. Anemia treatment with CERA in nondialyzed CKD patients may have significant potential to prevent or reduce cardiovascular complications in these patients. In an animal model, it was shown that chronic treatment with CERA protects against cardiac fibrosis [40]. However, a large-scale longitudinal clinical study should be conducted to determine whether the improvement of inflammation and endothelial dysfunction biomarkers caused by CERA treatment has an impact on better cardiovascular outcome in CKD patients. Further research may also determine whether CERA treatment already in early stages of CKD could improve the CVD outcome in these patients.

## 5. Conclusions

The results of our study indicate that all evaluated biomarkers of inflammation and endothelial dysfunction are significantly increased in nondialyzed CKD patients compared to controls. From our study, the most important new inflammatory biomarkers of CVD risk in CKD patients seem to be TNF-RI, sICAM-1, and MMP-9. CERA treatment by use of MPG-EPO has anti-inflammatory action, may diminish endothelial dysfunction, and improves left ventricle function in CKD patients. These beneficial effects of MPG-EPO treatment in these patients could be due to the correction of anemia and improvement of blood circulation and oxygenation of tissue and organs. CERA treatment could be considered as a novel therapeutic approach to prevent some mechanisms involved in pathogenesis of the atherosclerotic process and CVD in CKD patients. Further intensive research on large groups of patients is needed to confirm the positive correlation between CERA treatment and the decrease of cardiovascular complications in CKD patients.

## Figures and Tables

**Table 1:** Results of biochemical parameters in CKD patients and control group.

Biochemical parameters	CKD patients before treatment	CKD patients after treatment	Control group
	(*n* = 25) (Me; 25–75%)	(*n* = 25) (Me; 25–75%)	(*n* = 20) (Me; 25–75%)
Hb (g/dL)	9.1 (8.7–10.0)^*∗*^	11.6 (11.1–12.1)^*∗*°^	14.4 (13.3–14.9)
Creatinine (*µ*mol/L)	269 (233–340)^*∗*^	271 (223–348)^*∗*^	90 (77–108)
eGFR (MDRD) (mL/min/1.73 m^2^)	19.6 (15.5–22.8)^*∗*^	18 (15–24.2)^*∗*^	65 (63–74)
CRP (mg/L)	18 (14.1–22.8)^*∗*^	16 (12.2–20.4)^*∗*^	4.5 (3.7–6.9)
NT-proBNP (pg/mL)	355.6 (282.3–432.7)^*∗*^	302 (182.1–401.3)^*∗*°^	75 (49–101)

^*∗*^ *p* < 0.05 versus control group; °*p* < 0.05 versus CKD patients before treatment.

CKD = chronic kidney disease, (Me; 25–75%) = median and interquartile range, Hb = hemoglobin, eGFR (MDRD) = estimated glomerular filtration rate (Modification of Diet in Renal Disease equation), CRP = C-reactive protein, and NT-proBNP = N-terminal pro-brain natriuretic peptide.

**Table 2:** Results of assessed biomarkers in CKD patients and control group.

Biomarkers	CKD patients before treatment	CKD patients after treatment	Control group
	(*n* = 25) (Me; 25–75%)	(*n* = 25) (Me; 25–75%)	(*n* = 20) (Me; 25–75%)
IL-1*β* (pg/mL)	2.78 (2.00–3.36)^*∗*^	2.69 (2.48–3.15)^*∗*^	1.25 (0.89–1.65)
TNF-RI (pg/mL)	4644 (4156–5677)^*∗*^	4979 (4236–6667)^*∗*^	1151 (930–1332)
TNF-RII (pg/mL)	8010 (7298–8689)^*∗*^	9180 (8845–9908)^*∗*°^	2351 (1900–2600)
sFasL (pg/mL)	85.9 (68.0–109.9)^*∗*^	94.3 (75.6–103.8)^*∗*^	47.0 (30.5–80.0)
sFas (pg/mL)	3272 (2734–3799)^*∗*^	3206 (2826–3715)^*∗*^	465.0 (368.0–645.0)
sE-selectin (ng/mL)	28.3 (24.2–33.6)^*∗*^	33.5 (26.0–36.2)^*∗*^	15.5 (11.7–22.9)
sICAM-1 (ng/mL)	402.0 (336.0–504.0)^*∗*^	466.0 (355.0–518.0)^*∗*^	242.0 (234.0–272.0)
TIMP-1 (ng/mL)	203.0 (186.5–258.5)^*∗*^	212.0 (178.0–228.0)^*∗*^	110.0 (91.0–131.0)
sVCAM-1 (ng/mL)	2380 (1326–2790)^*∗*^	2600 (1720–4129)^*∗*°^	840.0 (760.0–1252.0)
MMP-9 (ng/mL)	1062 (665–1455)^*∗*^	586.0 (450.0–764.0)^*∗*°^	280.0 (217.0–292.0)
TGF-*β*1 (pg/mL)	21.4 (19.0–30.1)^*∗*^	21.2 (16.5–29.3)^*∗*^	11.6 (9.7–15.5)

^*∗*^ *p* < 0.05 versus control group; °*p* < 0.05 versus CKD patients before treatment.

CKD = chronic kidney disease, (Me; 25–75%) = median and interquartile range, IL-1*β* = interleukin 1*β*, TNF-RI = soluble tumor necrosis factor receptor I, TNF-RII = soluble tumor necrosis factor receptor II, sFasL = soluble Fas ligand, sFas = soluble Fas, sICAM-1 = soluble intercellular adhesion molecule-1, TIMP-1 = tissue inhibitor of metalloproteinase-1, sVCAM-1 = soluble human vascular cell adhesion molecule-1, MMP-9 = metalloproteinase-9, and TGF-*β*1 = transforming growth factor-beta 1.

**Table 3:** Correlations between serum Hb concentration and evaluated biomarkers in CKD patients.

Biomarkers	CKD patients before treatment	CKD patients after treatment
	*n* = 25	*n* = 25
	Hb	Hb
IL-1*β* (pg/mL)	−0.140	0.117
sTNF-RI (pg/mL)	−0.040	−0.444^*∗*^
sTNF-RII (pg/mL)	−0.157	0.270
sFasL (pg/mL)	−0.307	−0.015
sFas (pg/mL)	−0.196	−0.164
sE-selectin (ng/mL)	−0.025	−0.023
sICAM-1 (ng/mL)	0.148	−0.403^*∗*^
TIMP-1 (ng/mL)	−0.156	−0.222
sVCAM-1 (ng/mL)	0.116	0.083
MMP-9 (ng/mL)	0.102	−0.140
TGF-*β*1 (pg/mL)	−0.026	−0.138

Spearman *r* correlation coefficient values, ^*∗*^ *p* < 0.05.

Hb = hemoglobin, CKD = chronic kidney disease, IL-1*β* = interleukin 1*β*, TNF-RI = soluble tumor necrosis factor receptor I, TNF-RII = soluble tumor necrosis factor receptor II, sFasL = soluble Fas ligand, sFas = soluble Fas, sICAM-1 = soluble intercellular adhesion molecule-1, TIMP-1 = tissue inhibitor of metalloproteinase-1, sVCAM-1 = soluble human vascular cell adhesion molecule-1, MMP-9 = metalloproteinase-9, and TGF-*β*1 = transforming growth factor- beta 1.

**Table 4:** Correlation between increase of Hb concentration expressed as absolute values (Δ) or as percentages (%) and change of evaluated biomarkers expressed as absolute values (Δ) or as percentages (%) in CKD patients.

Biomarkers (Δ) after-before treatment	Hb (Δ) after-before treatment	Biomarkers (%) [(after-before)/before treatment] × 100	Hb (%) [(after-before)/before treatment] × 100
IL-1*β*	−0.511^*∗*^	IL-1*β*	−0.484^*∗*^
TNF-RI	−0.061	TNF-RI	−0.081
TNF-RII	**−**0.250	TNF-RII	−0.276
sFasL	−0.070	sFasL	−0.088
sFas	−0.096	sFas	−0.093
sE-selectin	−0.128	sE-selectin	−0.105
sICAM-1	0.144	sICAM-1	0.125
TIMP-1	0.040	TIMP-1	0.066
sVCAM-1	−0.074	sVCAM-1	0.013
MMP-9	0.094	MMP-9	0.055
TGF-*β*1	−0.124	TGF-*β*1	−0.098

Spearman *r* correlation coefficient values, ^*∗*^ *p* < 0.05.

Hb = hemoglobin, CKD = chronic kidney disease, IL-1*β* = interleukin 1*β*, TNF-RI = soluble tumor necrosis factor receptor I, TNF-RII = soluble tumor necrosis factor receptor II, sFasL = soluble Fas ligand, sFas = soluble Fas, sICAM-1 = soluble intercellular adhesion molecule-1, TIMP-1 = tissue inhibitor of metalloproteinase-1, sVCAM-1 = soluble human vascular cell adhesion molecule-1, MMP-9 = metalloproteinase-9, and TGF-*β*1 = transforming growth factor-beta 1.

**Table 5:** Echocardiographic parameters in CKD patients and control group.

	CKD patients before treatment	CKD patients after treatment	Control group
	(*n* = 25) (Me; 25–75%)	(*n* = 25) (Me; 25–75%)	(*n* = 20) (Me; 25–75%)
IVSd (mm)	16 (15.7–18.3)^*∗*^	15 (11.5–18.1)^*∗*^	12 (10.6–13.4)
LVM (g)	287.8 (217.7–357.9)^*∗*^	279.2 (183.2–355.2)^*∗*^	206 (163.8–248.2)
LVESd (mm)	41 (34.8–47.2)^*∗*^	39.9 (32.7–45.1)^*∗*^	36 (30.1–41.9)
LVEDd (mm)	48.5 (41.8–55.2)^*∗*^	47.1 (41.5–52.7)^*∗*^	40 (32.6–47.4)
LA (mm)	41.9 (39.2–44.6)^*∗*^	41.3 (37.1–43.5)^*∗*^	31 (28.8–33.2)
EF (%)	35 (30–42)^*∗*^	42 (35–48)^*∗*°^	60 (51–69)
LVH (%)	100^*∗*^	100^*∗*^	20
LVDD (%)	96.7^*∗*^	91.6^*∗*^	27

^*∗*^ *p* < 0.05 versus control group; °*p* < 0.05 versus CKD patients before treatment.

CKD = chronic kidney disease, IVSd = intraventricular septal diameter, LVM = left ventricular mass, LVESd = left ventricular end systolic diameter, LVEDd = left ventricular end diastolic diameter, LA = left atrial diameter, EF = left ventricular ejection fraction, LVH = left ventricular hypertrophy, and LVDD = left ventricular diastolic dysfunction.

**Table 6:** Correlation between serum Hb concentration and echocardiographic parameters of left ventricle structure and function in CKD patients.

Echocardiographic parameters	CKD patients before treatment	CKD patients after treatment
	(*n* = 25)	(*n* = 25)
	Hb	Hb
IVSd (mm)	−0.742	−0.425
LVM (g)	−0.953	−0.613
LVESd (mm)	−0.768	−0.543^*∗*^
LVEDd (mm)	−0.748	−0.350
LA (mm)	−0.774	−0.498
EF (%)	0.949	0.288^*∗*^

Spearman *r* correlation coefficient values, ^*∗*^ *p* < 0.05.

Hb = hemoglobin, CKD = chronic kidney disease, IVSd = intraventricular septal diameter, LVM = left ventricular mass, LVESd = left ventricular end systolic diameter, LVEDd = left ventricular end diastolic diameter, LA = left atrial diameter, and EF = left ventricular ejection fraction.
